# Somatic mosaicism of an intragenic *FANCB* duplication in both fibroblast and peripheral blood cells observed in a Fanconi anemia patient leads to milder phenotype

**DOI:** 10.1002/mgg3.350

**Published:** 2017-11-30

**Authors:** Rajalakshmi S. Asur, Danielle C. Kimble, Francis P. Lach, Moonjung Jung, Frank X. Donovan, Aparna Kamat, Raymond J. Noonan, James W. Thomas, Morgan Park, Peter Chines, Adrianna Vlachos, Arleen D. Auerbach, Agata Smogorzewska, Settara C. Chandrasekharappa

**Affiliations:** ^1^ Cancer Genetics and Comparative Genomics Branch National Human Genome Research Institute NIH Bethesda MD USA; ^2^ Laboratory of Genome Maintenance The Rockefeller University New York NY USA; ^3^ NIH Intramural Sequencing Center National Human Genome Research Institute NIH Rockville MD USA; ^4^ Medical Genomics and Metabolic Genetics Branch National Human Genome Research Institute NIH Bethesda MD USA; ^5^ Hematology/Oncology and Stem Cell Transplantation Cohen Children's Medical Center New Hyde Park NY USA; ^6^ The Feinstein Institute for Medical Research of Northwell Health Manhasset NY USA; ^7^ Human Genetics and Hematology Program The Rockefeller University New York NY USA

**Keywords:** droplet digital PCR, *FANCB*, intragenic duplication, milder phenotype, revertant mosaicism

## Abstract

**Background:**

Fanconi anemia (FA) is a rare disorder characterized by congenital malformations, progressive bone marrow failure, and predisposition to cancer. Patients harboring X‐linked *FANCB* pathogenic variants usually present with severe congenital malformations resembling VACTERL syndrome with hydrocephalus.

**Methods:**

We employed the diepoxybutane (DEB) test for FA diagnosis, arrayCGH for detection of duplication, targeted capture and next‐gen sequencing for defining the duplication breakpoint, PacBio sequencing of full‐length FANCB aberrant transcript, FANCD2 ubiquitination and foci formation assays for the evaluation of FANCB protein function by viral transduction of FANCB‐null cells with lentiviral *FANCB* WT and mutant expression constructs, and droplet digital PCR for quantitation of the duplication in the genomic DNA and cDNA.

**Results:**

We describe here an FA‐B patient with a mild phenotype. The DEB diagnostic test for FA revealed somatic mosaicism. We identified a 9154 bp intragenic duplication in *FANCB,* covering the first coding exon 3 and the flanking regions. A four bp homology (GTAG) present at both ends of the breakpoint is consistent with microhomology‐mediated duplication mechanism. The duplicated allele gives rise to an aberrant transcript containing exon 3 duplication, predicted to introduce a stop codon in FANCB protein (p.A319*). Duplication levels in the peripheral blood DNA declined from 93% to 7.9% in the span of eleven years. Moreover, the patient fibroblasts have shown 8% of wild‐type (WT) allele and his carrier mother showed higher than expected levels of WT allele (79% vs. 50%) in peripheral blood, suggesting that the duplication was highly unstable.

**Conclusion:**

Unlike sequence point variants, intragenic duplications are difficult to precisely define, accurately quantify, and may be very unstable, challenging the proper diagnosis. The reversion of genomic duplication to the WT allele results in somatic mosaicism and may explain the relatively milder phenotype displayed by the FA‐B patient described here.

## 1 INTRODUCTION

Fanconi anemia (FA) is a DNA repair deficiency syndrome, and is characterized by developmental abnormalities, bone marrow failure, and predisposition to cancer, including myelodysplastic syndrome (MDS), acute myeloid leukemia (AML) and solid tumors, especially squamous cell carcinoma of the head and neck (Kottemann & Smogorzewska, 2013; Mamrak, Shimamura, & Howlett, 2017; Mehta & Tolar, 2017). Apart from X‐linked *FANCB,* and dominant negative variants in *RAD51/FANCR*, FA is an autosomal recessive disease, caused by the inheritance of biallelic mutations in any of the 20 other FA genes: *FANCA, FANCC, FANCD1/BRCA2, FANCD2, FANCE, FANCF, FANCG, FANCI, FANCJ/BRIP1, FANCL, FANCM, FANCN/PALB2, FANCO/RAD51C, FANCP/SLX4, FANCQ/XPF, FANCS/BRCA1, FANCT/UBE2T, FANCU/XRCC2, FANCV/REV7, FANCW/RFWD3* (Knies et al., 2017; Mamrak et al., 2017; Wang & Smogorzewska, 2015). FA is both genetically and phenotypically a heterogeneous disorder. The underlying problem is inability of FA cells to repair DNA interstrand crosslinks (ICLs).

The congenital abnormalities in an FA patient may affect multiple organ systems and include growth (short stature), skin pigmentation, thumb and radial bone, uro‐genital, eye, ear and hearing, gastrointestinal, and central nervous system (CNS) anomalies (Auerbach, 2009). The phenotypic expression of these abnormalities is highly variable, and is displayed by about two‐thirds of FA patients. However, the malformations are usually severe in FA‐B patients. In fact, presentations of X‐linked VACTERL with hydrocephalus syndrome resulting in vertebral anomalies (V), anal atresia (A), cardiac anomalies (C), tracheoesophageal fistula (T), esophageal atresia (E), renal structural anomalies (R) limb anomalies (L), and hydrocephalus (H) overlap with those displayed by FA‐B patients (Alter & Rosenberg, 2013; Holden et al., 2006; McCauley et al., 2011; Mikat et al., 2016; Umana, Magoulas, Bi, & Bacino, 2011; Winberg et al., 2014). However, a positive FA diagnostic test, with increased chromosomal breakage in patient cells upon exposure to DNA crosslinking agents diepoxybutane (DEB) or mitomycin C (MMC), distinguishes FA‐B patients from the VACTERL patients.

Somatic (or revertant) mosaicism has been observed in nearly 25% of FA patients (Auerbach, 2009). It manifests itself by the appearance of a population of blood cells that lost the crosslink sensitivity and now appear WT. Mosaicism can be caused by the reversion of an inherited mutation or the introduction of a de novo variant with a consequence of reducing or eliminating the deleterious nature of the inherited variant (Gregory et al., 2001; Gross et al., 2002; Hamanoue et al., 2006; Lo Ten Foe et al., 1997; Mankad et al., 2006; Waisfisz et al., 1999).

We report here an FA patient who harbors an intragenic duplication in *FANCB* (OMIM: 300515) and displays a mild disease phenotype. We suggest that this unusual phenotype for an FA‐B patient is due to mosaicism caused by the disappearance of the duplication.

## 2 MATERIALS AND METHODS

### 2.1 Ethical compliance

This study was conducted with the approval of the Institutional Review Board at The Rockefeller University. Written informed consent was obtained from the legal guardians of the patient, in accordance with the Declaration of Helsinki. The Office of Human Subjects Research at the National Institutes of Health and the Institutional Review Board of the National Human Genome Research Institute approved the analysis of the molecular variants, from the cell lines and DNA.

### 2.2 Cell culture, DNA and RNA extraction

The lymphoblastoid cell lines (LCL) from the proband and his mother, established by Epstein–Barr virus (EBV)‐transformed B‐lymphocytes, were cultured in RPMI (Roswell Park Memorial Institute 1640) medium (Gibco, Paisley, UK), supplemented with 20% fetal bovine serum (FBS) (Sigma‐Aldrich, St. Louis, MO), penicillin–streptomycin (100 U/ml penicillin G sodium and 100 μg/ml streptomycin in 0.85% saline) (Gibco), 2.5 μg/ml amphotericin B (Gibco) and 2 mM L‐glutamine (Gibco). The cells were grown in a fully humidified incubator with 5% CO_2_ at 37°C. Primary skin fibroblasts from the proband were grown in Dulbecco Modified Eagle medium (DMEM, GIBCO) supplemented with 15% FBS (Sigma‐Aldrich, MO) and further supplemented and cultured as above. Control LCL (GM 18507) and Fibroblast (HGADFN168) cell lines were also grown as described above. Genomic DNA from the lymphoblast and fibroblast cell cultures was extracted using the DNeasy Blood and Tissue Kit (Qiagen, Valencia, CA). Total RNA was extracted using the RNeasy Mini Kit (Qiagen) with an in‐column DNase treatment. Five micrograms of RNA was then reverse transcribed using the SuperScript™ IV First‐Strand Synthesis System (Invitrogen, Grand Island, NY). The cDNA synthesis was performed per the manufacturer's protocol. The cDNA was then used as a template for PCR.

### 2.3 Diagnostic DEB testing

Initial diagnostic testing for chromosomal breakage induced by DEB was performed on the proband's primary fibroblasts (Fib‐2005) and peripheral blood cells (PB‐2005) isolated from the patient in 2005 as described earlier (Auerbach, 2015). To follow the progression of mosaicism over time, chromosomal breakage was again evaluated using a new blood sample drawn from the patient in 2016 (PB‐2016) and the LCL derived from that blood (LCL‐B). The proband's LCL derived from the PB drawn in 2005 (LCL‐A) was also studied.

### 2.4 aCGH

Agilent CGH array was designed to query the entire length and extended regions of all FA and other inherited bone marrow failure syndrome genes. The *FANCB* (ENSG00000181544.13; ENST00000398334.5; NM_001018113.2) gene region on the array extended to 150 kb on either side of the gene interval. The experimental procedures are as described previously (Flynn et al., 2014). Briefly, genomic DNA from the patient and a reference male DNA were differentially labeled and hybridized to the array.

### 2.5 Next‐gen sequencing (NGS) and analysis

Targeted capturing and sequencing of the entire length of FA genes were performed as described previously (Chandrasekharappa et al., 2013). The number of reads that mapped to 99 bp intervals across the *FANCB* gene were tallied with the BedTools command, coverageBed, and plotted for a randomly selected sample as a control and the samples with the duplication (proband fibroblast and proband LCL) in the UCSC genome browser (hg19) with BedGraph tracks. The precise location of the duplication (chrX:14877976‐14887129 or chrX:14877972‐14887133, as there is microhomology at the edges of the duplication) was determined based on a combined manual analysis of the read depth, clustering of soft‐clipped reads, and reads with unmapped mate‐pairs. Reads from the *FANCB* region were re‐mapped to an altered sequence using Novoalign including the inferred duplication to confirm the breakpoints of the duplication.

### 2.6 PCR assay for the FANCB duplication breakpoint junction in genomic DNA

The gDNA samples were amplified using the KAPA2G Fast HotStart ReadyMix PCR Kit (KAPA Biosystems Inc., Wilmington, MA). The reaction mix and amplification conditions were set‐up as per the manufacturer's instructions. The sequence for the primers used for the amplification of the 702 bp *FANCB* duplication junction product were as follows‐ forward primer 5′ GGGTTGCTCAAAGGGTACAA 3′ (chrX:14886733‐14886752) and reverse primer 5′ TCAGGGAAAAGGGGGATTT 3′(chrX:14878265‐14878283). The *FANCB* duplication junction primers were designed such that they face each other across the duplication junction. We used primers to amplify a 91 bp in the intron 1 region of *FANCB* as control. The primer sequences were‐ forward 5′ CCAAGGATACTTTCTCTTGGTC 3′ (chrX:14888971‐14888992) and reverse 5′ CAGTGTGGTTCCAGATTATCC 3′ (chrX:14888902‐14888922). The universal M13 forward and reverse sequences were added as a tag to our primer sequence.

### 2.7 Sanger sequencing the PCR products from the FANCB duplication junction PCR in gDNA

The PCR products were treated with USB ExoSAP‐IT (Affymetrix, Santa Clara, CA) and sequencing reactions were carried out with the M13 forward (5′ tgtaaaacgacggccagt 3′) and reverse (5′ caggaaacagctatgacc 3′) primers, using the Bigdye Terminator v3.1 Cycle Sequencing kit (Applied Biosystems) and run on ABI3730XL sequencer.

### 2.8 Droplet Digital PCR (ddPCR) for quantitation of FANCB duplication junction in gDNA

Copy number variance analysis was performed using the ddPCR system (Bio‐Rad, Hercules, CA), adhering to the MIQE guidelines (Huggett et al., 2013). The primers and FAM‐labeled probe for the *FANCB* gDNA duplication junction were custom designed to target a 185 bp unique region and are as follows‐ forward primer sequence 5′ ATCTGGGACAATAGGCATCA 3′, reverse primer sequence 5′ TAGGACCCCTCACCTGTGTA 3′ and probe sequence AAGATAGGGTAGACACCTTAAGAGCTC. The probe was designed to hybridize to the *FANCB* exon 3‐3 duplication junction in the gDNA of our samples. The HEX‐labeled fluorescent probe hybridized to an amplicon targeting 60 bp of intron 22i in the *AP3B1* gene, which served as a 2‐copy reference in each cell (*AP3B1* ddPCR copy number assay, UniqueAssayID: dHsaCP2500348, Bio‐Rad). The PCR reaction mix was prepared per the manufacturer's instructions, with 22 ng gDNA in a total reaction volume of 22 μl, of which 20 μl was partitioned into 13,000‐20,000 water‐in‐oil droplets using the Automated Droplet Generator (Bio‐Rad). The optimal primer annealing temperature was determined by gradient PCR and subsequently, samples were amplified on the C1000 Touch Thermal Cycler with the optimal annealing temperature of 58°C (Bio‐Rad). After amplification, the 96‐well PCR plate was placed on the QX200 Droplet Reader (Bio‐Rad) and the data were analyzed using the QuantaSoft analysis software (version 1.7.4). The QuantaSoft software was then used to calculate the copy number variance (CNV) in our sample. CNV is determined by calculating the ratio of the target (*FANCB* duplication junction) molecule concentration to the ratio of the reference (*AP3B1*) species concentration multiplied by the number of copies of the reference species (2 copies of *AP3B1*) in the genome. In the absence of the duplication junction in a given droplet, no PCR amplification of the *FANCB* exon 3‐3 duplication product would take place. Only wells with >13,000 droplets generated were analyzed. The average CNV for each sample was calculated from nine observations across three experimental replicates.

### 2.9 RT‐PCR, gel extraction, and cloning of the full‐length FANCB transcript with the duplication

PCR was performed using cDNA as a template with the KOD Xtreme™ Hot Start DNA Polymerase (EMD Millipore, Billerica, CA). The forward primer 5′ TTGGAGCAGATG GATACCGT 3′ (chrX:14891147‐14891166) and reverse primer 5′ AAAGTTTCTACTACAGTAAGCCTCG 3′ (chrX:14861567‐14861591) were used to amplify the full‐length *FANCB* transcript. This generates two products, a wild‐type 2952 bp product and a 3973 bp product with the exon 3‐3 duplication. The PCR mix was set‐up as per the manufacturer's protocol. The larger 3.9 kb product was used as a template for PacBio sequencing, following gel extraction and cloning.

The sequence of the *FANCB* aberrant transcript was obtained by PacBio sequencing and primers were subsequently designed to amplify the unique 1009 bp RT‐PCR product from the proband's fibroblasts using the forward primer 5′ TTGGAGCAGATGGATACCGT 3′ (chrX:1489 1147‐14891166) and the reverse primer 5′TCAAATGCAAATTGATTCCAGT 3′ (chrX: 14883667 ‐14883688). This represents the coding region of the aberrant transcript. Following gel extraction and cloning, the 2.9 kb wild‐type and the 1009 bp aberrant *FANCB* transcripts were used for viral transduction to generate expression constructs.

The RT‐PCR products were excised from the gel and the DNA was extracted using the PureLink^®^ Quick Gel Extraction Kit (Invitrogen). The extraction was performed per the manufacturer's protocol and the resulting DNA was concentrated by ethanol precipitation. The CloneJet PCR cloning kit (Thermo Fisher Scientific, Waltham, MA) was used to clone 250 ng of the concentrated DNA obtained above, into a positive selection cloning pJET1.2/blunt vector. The individual colonies obtained were then screened by PCR for insertion of the correct size product.

### 2.10 PacBio Sequencing of full‐length FANCB aberrant transcript

The Pacific Biosciences (Menlo Park, CA) protocol “Preparing SMRTbell Libraries using PacBio Barcoded Universal Primers for Multiplex SMRT Sequencing” was used to create a PacBio sequencing library from the pooled amplicons using only the library construction portion of the protocol. The resulting library size and purity was assessed using a Bioanalyzer (Agilent). Sequencing was performed on an RS II sequencer (Pacific Biosciences) using P6‐C4 sequencing chemistry with 240 min movies. Two SMRT cells were used to generate over 126,000 polymerase reads. PacBio raw reads were converted into circular consensus sequences (CCS) using PacBio SMRTanalysis software (version 2.3). CCS were analyzed using the PacBio ToFu pipeline (version 2.2.3) (Gordon et al., 2015), to generate polished transcript sequences. The transcripts were aligned to a reference using GMAP (version 2014‐12‐21) (Wu & Watanabe, 2005).

### 2.11 Droplet Digital PCR for quantitation of FANCB exon 3‐exon 3 junction in cDNA

The cDNA from the proband, mother, and control samples were used as template to quantify the expression of the unique *FANCB* exon 3‐exon 3 junction using the Bio‐Rad ddPCR system. The reaction mix was set‐up as per the manufacturer's instructions, and PCR was performed with an annealing temperature of 54°C. The primer and probe sequence for the *FANCB* duplication junction in cDNA were custom designed and labeled with FAM. The forward primer sequence is 5′ ATTCAGGTGGAGGAAACCT 3′, the reverse primer sequence is 5′ CGTTAGATGACATTGCTTG 3′ and the probe sequence is TGCTTGTGCTGTATGGAAAGA. The HEX labeled *FANCB* exon 4‐exon 5 primer and probe (UniqueAssayID: qHsaCIP0039596, Bio‐Rad) was used as a reference. The QuantaSoft software was then used for rare event detection (RED) in our samples. RED is determined by calculating the ratio of the target (*FANCB* duplication junction) molecule concentration to the ratio of the reference (*FANCB* control) molecule concentration in the samples. The ratio was then multiplied by 100 in order to obtain the percentage of the duplication junction in our cDNA samples.

### 2.12 Viral transduction

For lentiviral transduction experiments, the wild‐type and aberrant *FANCB* transcripts were transferred from pJET1.2 to pHAGE CMV N‐terminus HA‐FLAG vector using Gateway recombination (Thermo Fisher Scientific). These were packaged in HEK293T cells using Trans‐IT transfection reagent per the manufacturer's protocol (Mirus) and were delivered using lentiviral transduction into proband fibroblasts, FANCB mutant (null) fibroblasts, and BJ foreskin normal control fibroblasts (ATCC) that were transformed by the expression of HPV16 E6E7 by retroviral infection.

### 2.13 Mitomycin C (MMC) treatment to assess FANCD2 ubiquitination

Patient fibroblasts, patient LCL, transformed proband fibroblasts, and FANCB mutant cells were seeded 24 hr before treatment. The next day, the cells were treated with 1 μM MMC for 24 hr or were left untreated. After 24 hr, cells were harvested using 0.25% Trypsin‐EDTA (Gibco).

### 2.14 Western blotting

Whole‐cell extracts were prepared by performing cell lysis using 2× Laemmli sample buffer (Bio‐Rad), followed by sonication and boiling. Samples were separated on 3%–8% Tris‐Acetate or 4%–12% Bis‐Tris gradient gels (Invitrogen) by SDS‐PAGE. Membranes were blocked with 5% skim milk in 0.1% Tween 20/TBS (TBST) for 1 hr. Immunoblot was performed using primary antibodies against FANCD2 (Novus NB100‐182), HA.11 (BioLegend 16B12) and α‐Tubulin (Sigma DMA1). Membranes were incubated with primary antibodies at room temperature for 2 hr then washed three times with TBST. Membranes were incubated with anti‐rabbit or anti‐mouse HRP‐linked secondary antibodies at room temperature for 1 hr and washed three times with TBST. Membranes were developed using Western Lightning Plus‐ECL (PerkinElmer) and images were obtained by chemiluminescence on ImageQuant LAS4000 (General Electric).

### 2.15 Immunofluorescence for FANCD2 foci formation

Cells were seeded onto cover slides in 6‐well culture dishes. The next day, cells were treated with 1 μM MMC for 24 hr or were left untreated, after which cells were fixed with 3.7% formaldehyde and permeabilized with 0.5% Triton in PBS. Cells were blocked in 5% FBS in PBS (w/v) for 1 hr at room temperature, incubated with anti‐rabbit FANCD2 antibody (Novus NB100‐182) overnight at 4°C, and washed three times with 5% FBS in PBS. Cells were then incubated with Alexa 488 goat anti‐rabbit IgG (H + L) (Invitrogen #A32731) for 1 hr at room temperature, washed three times in 5% FBS in PBS, then coverslips were mounted using DAPI Fluoromount‐G® (SouthernBiotech).

## 3 RESULTS

### 3.1 Clinical presentation

The patient presented at 19 months of age with failure‐to‐thrive. The birth weight was 3.12 kg and he was at the 50^th^ percentile for height and weight at 6 months of age, but at presentation his height was at the 5‐10^th^ percentile and his weight was below the 5^th^ percentile. He was referred to Gastroenterology and Hematology for evaluation and the possibility of the diagnosis of an inherited bone marrow failure syndrome. He had no symptoms of diarrhea and no previous history of infections but had delay in speech development. The physical exam was normal except for several hyperpigmented areas on the abdomen and the axilla, and hypopigmented areas on the back and legs. Sweat testing was negative. Initial blood counts revealed mild neutropenia (Table 1). The bone marrow biopsy showed a few areas with 50‐70% cellularity, formed mainly by erythroid cells; normal myeloid maturation; and adequate number of megakaryocytes. Cytogenetics and FISH were normal.

**Table 1:** Hematological manifestations in the proband

	Patient Age		
	19 months	9 years	13 years
Blood Count			
WBC (K/μl)	5.36	3.7	3.6
ANC (cells/μl)	1288	1500	1000
Hb (g/dl)	12.2	13.5	12.9
MCV (fl)	82.6	86.3	90.2
Platelets (K/μL)	258	155	140

The patient is presently 13 years of age and is at the 16^th^ percentile for height and less than the 5^th^ percentile for weight. He has mild neutropenia and thrombocytopenia and continues to be borderline macrocytic. The bone marrow aspirate and biopsy at age 13 showed hypocellular marrow (20%) with trilineage hematopoiesis with maturation, focal areas of erythroid predominance and decreased megakaryocytes; no dysplasia; and, markedly decreased to absent iron stores. Cytogenetics and FISH were normal. He has no significant health issues at this time.

### 3.2 DEB test and complementation analysis reveal diagnosis of FA‐B group with revertant mosaicism

**Figure 1:**
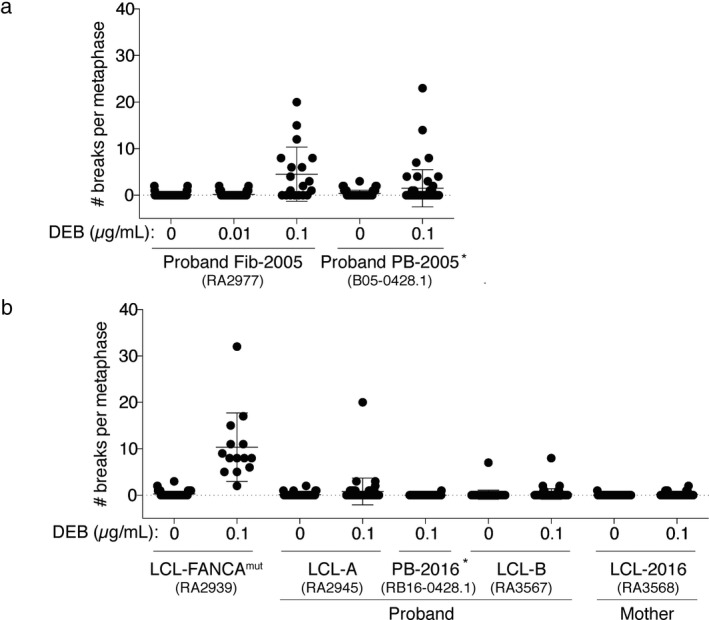
Results of chromosome breakage with and without DEB. (a) Results of clinical testing. Proband fibroblasts (Fib‐2005) and PB‐2005 were tested as part of the original diagnosis in the year 2005. (b) Results of research testing performed in 2017 on the proband's LCL from 2005 (LCL‐A) and 2016 (LCL‐B), as well as the proband's peripheral blood drawn in 2016 (PB‐2016). The FANCA ^mut^ LCL represents the typical DEB test results from the LCL of a patient with FA and the mother's LCL (LCL‐2016) was included as a healthy control. The details of the breakage test results are shown in Table S1 Asterisks indicated that the test was done on a separate date even though it is shown on the same graph

Clinical DEB breakage test using proband's dermal fibroblasts (Fib‐2005; RA2977) was consistent with Fanconi anemia (Figure 1, Table S1), albeit a high dose of DEB (0.1 μg/ml) was necessary to elicit breakage levels seen in typical FA patients. Peripheral blood at the time of diagnosis (PB‐2005; B05‐0428.1) exhibited elevated chromosomal breakage. However, only thirty percent of cells analyzed at that time showed breaks, consistent with revertant mosaicism in the patient cells. The presence of mosaicism was confirmed in samples collected later from the patient, including the peripheral blood from 2016 (PB‐2016) which had an average of 0.01 breaks per metaphase and the LCL derived from this PB (LCL‐B), which showed an average of 0.28 breaks per metaphase (Figure 1b). In addition, the LCL cell line established from PB collected at diagnosis in 2005 (LCL‐A), showed an average of 0.82 metaphases with breaks when tested in 2017. Taken together, the breakage studies on different samples from the patient, suggested presence of an unstable mutant allele that was being reverted both during in vivo and in vitro growth. Complementation tests performed using the patient fibroblasts showed correction of the cellular sensitivity to crosslinking agents by the expression of *FANCB*.

### 3.3 Molecular diagnosis reveals that the patient carries an intragenic duplication in the FANCB gene

**Figure 2:**
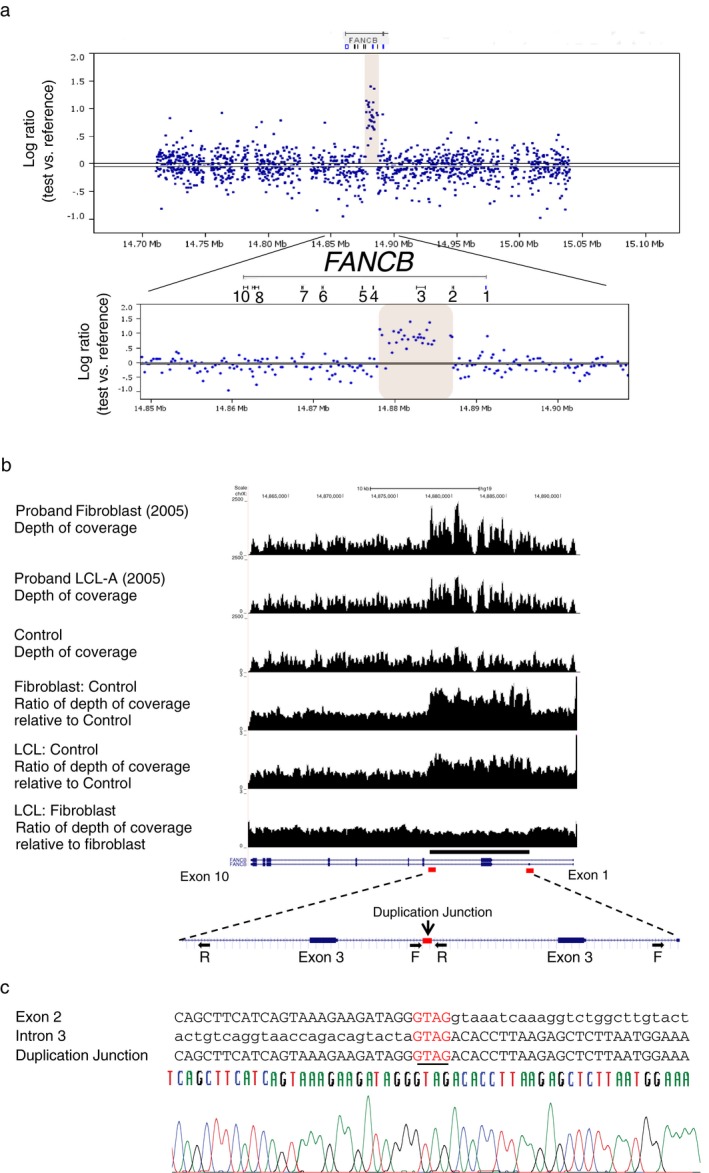
Detection and sequencing of duplication breakpoint. (a) ArrayCGH of DNA from proband's fibroblasts. Differential hybridization data obtained from the hybridization of genomic DNA from proband fibroblasts against a male reference DNA on a custom designed CGH array. The Log2 ratio of the signal intensity from test versus reference DNA is shown on the *y*‐axis, and X‐ axis shows the entire *FANCB* gene region. Increased signal intensity from proband DNA (expanded at the bottom), around exon 3 indicates duplication of this ~10 kb region in *FANCB* (NM_001018113.2). (b) Targeted capture and next‐gen sequencing (NGS) of the *FANCB* gene region. Targeted capture/sequencing of genomic DNA shows increased number of reads for the duplicated region (chrX:14877976‐14887129 or chrX:14877972‐14887133) in the proband fibroblast and LCL‐A‐2005, compared to a control DNA (top 3 tracks). The ratio of depth of coverage (bottom 3 tracks) confirms the duplicated region. Discordant reads at the region of duplication showed a tandem intragenic duplication of 9154 bp in *FANCB*, head to tail, in the same orientation and is expanded at the bottom of the hg tracks for *FANCB*. The red rectangular boxes indicate a shared homology of four bases (GTAG) at both ends of the breakpoint region, but only one in the duplicated region. (c) Sequence alignment of the *FANCB* duplication junction. The sequence alignment of the breakpoint junction, along with the sequence of the exon 2 and intron 3, the two ends of the duplication are shown here. The sequence at the junction suggested a microhomology‐mediated duplication event leading to the disease

Conventional Sanger sequencing of the coding region and NGS of the entire *FANCB* gene did not reveal any pathogenic sequence variants. However, aCGH designed to query the entire length of all FA genes showed an increased signal indicating a duplication of an intragenic region of *FANCB* in DNA from the patient's fibroblasts (Figure 2a). The NGS data showed a clear increase in the number of sequence reads for the same intragenic *FANCB* region. The sequence reads crossing the breakpoint provided the precise boundaries of the duplication and revealed a tandem intragenic duplication of 9154 bp in *FANCB* (Figure 2b). The duplicated region (chrX:14877976‐14887129 or 14877980‐14887133) extended from within the alternatively spliced, noncoding exon 2, the entire exon 3, and nearly 90% of intron 3. A shared homology of four bases GTAG (CTAC in the coding strand) at both ends of the breakpoint region (chrX:14877972‐14877976 at the proximal end and chrX:14887129‐14887133 at the distal end), but only one in the duplicated region, suggested a microhomology‐mediated duplication event leading to the disease (Figure 2b,c).

### 3.4 Quantitation of the FANCB duplication in genomic DNA reveals variable extent of duplication

**Figure 3:**
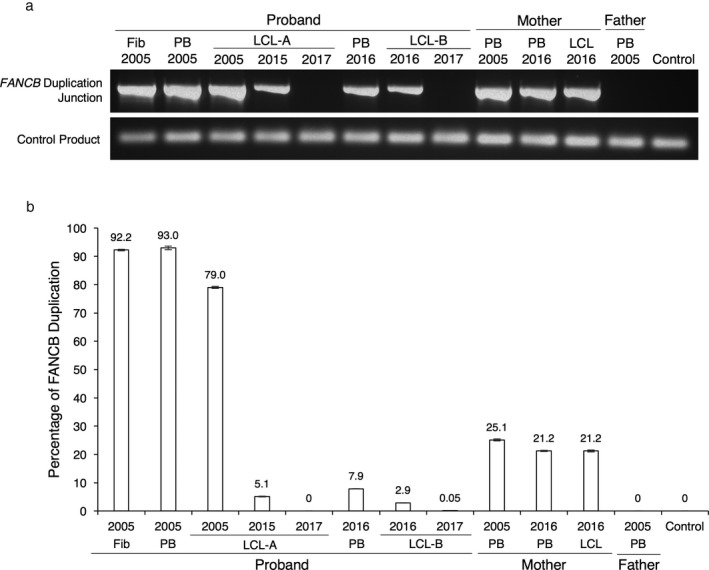
Evaluation of gDNA by PCR to show the presence of the unique duplication junction in the proband and the mother. (a) PCR of *FANCB* duplication junction. DNA samples from the patient, mother, father, and control were tested for the presence of the breakpoint junction. Presence of the duplication in the mother's as well as the proband's DNA was observed. The top panel represents the 702 bp PCR product of the *FANCB* duplicated region. The bottom panel represents a 91 bp PCR product from the *FANCB* intron 1 (control) amplification. Fib refers to Fibroblast DNA and PB refers to peripheral blood DNA. LCL‐A was established from the patient's PB‐2005, and DNA was collected from the LCL at that time (LCL‐A‐2005). This cell line was subsequently grown at different times in 2015 and 2017. LCL‐B was established from the patient's PB‐2016, and DNA was collected at that time (LCL‐B‐2016). This cell line was grown again in 2017 and DNA was collected (LCL‐B‐2017). (b) Copy Number Variance Analysis of *FANCB* duplication in gDNA using ddPCR. Standard errors are barely visible as they do not exceed ±0.63%; *n* = 9 observations per sample. The copy number variance was evaluated by performing ddPCR with probes specific for the *FANCB* genomic DNA duplication junction region, and a two‐copy reference gene. The variability in the extent of duplication in DNA from proband supports mosaicism. The extent of duplication in DNA from mother is <50% indicating mosaicism in her DNA as well

A PCR assay was designed to amplify the breakpoint junction in the gDNA. The presence of the junction and thus the duplication was apparent in DNA from the proband and mother, and as expected for a X‐linked disease, not in the DNA from father (Figure 3a). A ddPCR assay was developed to quantitate the extent of duplication in the DNA samples. The number of copies of the duplication junction relative to a 2‐copy control from an unrelated position in the gDNA was quantitated for the patient LCL, fibroblast and PB samples, as well as the mother's LCL and PB samples (Figure 3b). The DNA from the proband's fibroblasts had 92% duplication. The PB‐2005 DNA showed 93% duplication, whereas the LCL derived from this PB (LCL‐A) showed a decrease in the extent of duplication at three different time points over several years, with 79%, 5.1%, and 0% duplication in DNA extracted from cells grown in 2005, 2015, and 2017, respectively. The PB DNA collected eleven years later (PB‐2016), displayed 7.9% duplication, whereas the LCL generated from this PB (LCL‐B), displayed 2.9% and 0.05% duplication in DNA extracted from cells in 2016 and 2017, respectively.

We also quantitated the duplication in two PB DNA samples from the mother. PB‐2005 showed 25% duplication, whereas PB‐2016 and the LCL established from it (LCL‐2016) both showed 21% duplication. The observed duplication frequency in the mother's DNA was much less than the 50% anticipated for a heterozygous carrier, indicative of revertant mosaicism in the hematopoietic cells of the mother as well (Figure 3b).

### 3.5 The intragenic duplication results in a longer, aberrant FANCB transcript

**Figure 4:**
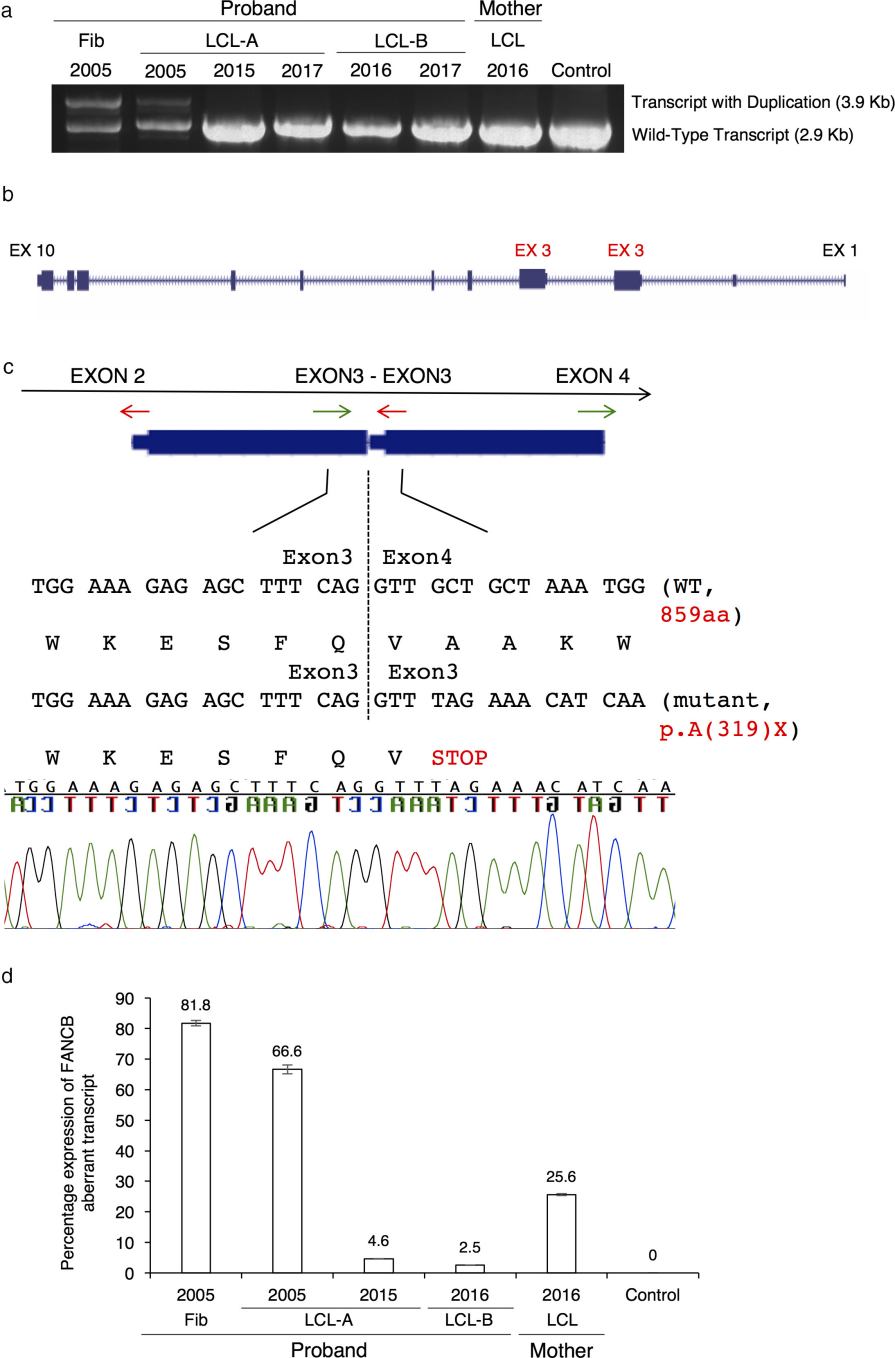
Evaluation of the consequence of duplication on the *FANCB* transcript. (a) RT‐PCR product of full‐length *FANCB* (NM_001018113.2) transcript in the proband, mother and control cells. The primers amplified a 2.9 kb *FANCB* transcript and a larger (3.9 kb) transcript with the duplication. Proband's cells, particularly the LCL‐A‐2005 and Fib‐2005, showed a larger transcript in addition to the WT transcript. (b) PacBio sequencing of the larger transcript. The sequences from the larger transcript, aligned to the reference genome modified to accommodate exon 3 duplication, indicate the duplicated exon 3 is now part of the aberrant transcript. (c) Diagram showing the unique *FANCB* exon 3‐exon 3 duplication junction. RT‐PCR and sequencing of the exon 3‐exon 3 junction region in the transcript with the duplication reveals a stop codon at the beginning of the junction. Primers (red and green arrows) were designed to amplify a unique 187 bp junction product from cDNA template. The transcript sequence predicts translation termination in close proximity to the junction. (d) The RNA from fibroblasts and LCL were evaluated by RT‐PCR followed by ddPCR quantitation, with probes specific for the aberrant *FANCB* message (probe for the exon 3‐exon 3 duplication junction region), and WT *FANCB* transcript (probe for the exon 4‐exon 5 junction). The percentage of aberrant transcript compared to the total *FANCB* transcript is presented. Vertical bars represent the standard error of the measurements; *n* = 3 separate observations performed in triplicate (total of nine data points) for each sample

We evaluated the consequence of the intragenic duplication on the *FANCB* transcript by RT‐PCR with primers amplifying the full‐length cDNA *FANCB* template (Figure 4a). The RNA from the proband LCL‐A‐2005 and fibroblasts (Fib‐2005) showed the presence of the full‐length wild‐type 2.9 kb product, as well as a 3.9 kb product that contains the duplication. Cloning and PacBio sequencing of the aberrant larger transcript revealed duplication of exon 3 (Figure 4b) and a unique exon 3‐exon 3 duplication junction sequence in the transcript (Figure 4c). The duplication of exon 3 results in generating a stop codon right near the junction, and if expressed, encodes a truncated protein p.A319*, much shorter than the full‐length protein of 859 amino acids.

### 3.6 The aberrant FANCB transcript level correlates with the level of genomic DNA duplication

We quantified the percentage of the transcript with the duplication compared to the wild‐type *FANCB* transcript using a probe specific for the duplication junction sequence (exon 3‐exon 3) and another probe specific for a *FANCB* wild‐type (exon 4‐exon 5) sequence (Figure 4d). We observed nearly 82% duplicated transcript in the proband fibroblast, whereas the proband LCL‐A‐2005, LCL‐A‐2015, and LCL‐B‐2016 samples displayed 66.6%, 4.6%, and 2.5% duplicated transcript, respectively. The mother's LCL exhibited a 25.6% duplicated message. The expression levels of the duplication in the *FANCB* transcript appears to be consistent with the CNV observed in the gDNA in these cells. Therefore, normal splicing of the duplicated transcript resulting in a wild‐type mRNA, if occurring, is minimal. Since the LCL‐A‐2017 and LCL‐B‐2017 genomic DNAs showed almost no duplication, we did not quantify the duplicated transcript in these samples by ddPCR.

### 3.7 FANCD2 ubiquitination is defective in the proband fibroblasts, but is regained in the proband LCL

**Figure 5:**
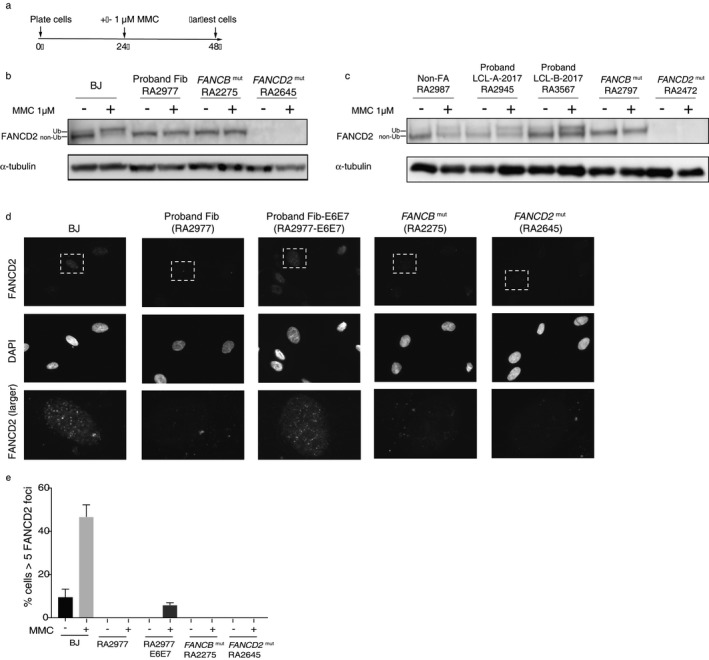
Functional assessment of Fanconi anemia pathway. (a) The experimental scheme for MMC treatment. Twenty‐four hours after plating, cells were cultured with or without MMC 1 μM for an additional 24 hr, after which the cells were harvested for western blot or immunostaining. (b) Western blot with FANCD2 antibody of BJ, proband fibroblasts, *FANCB* mutant (null) fibroblasts and *FANCD2* mutant (null) fibroblasts. (c) Western blot with FANCD2 antibody of non‐FA control lymphoblasts (LCL), proband LCL‐A‐2017, proband LCL‐B‐2017, *FANCB* mutant (null) LCL, and *FANCD2* mutant (null) LCL. (d) Representative figures of FANCD2 foci formation in the indicated cells. (e) Quantification of FANCD2 foci formation following treatment with or without 1 μM MMC. Experiments were performed in triplicate. One hundred cells were counted for each experiment

Proband fibroblasts showed the absence of FANCD2 ubiquitination either with or without MMC treatment, confirming the defective FA pathway function upstream of FANCD2 ubiquitination in this patient (Figure 5a–c). FANCD2 foci formation assay also showed the absence of detectable MMC‐induced FANCD2 foci (Figure 5d,e). On the other hand, both LCL‐A‐2017 and LCL‐B‐2017 showed ubiquitination of FANCD2 that was enhanced with MMC treatment (Figure 5c). This finding suggests that majority of these LCLs grown in 2017 were derived from revertant cells that have restored the intact FA pathway allowing ubiquitination of FANCD2 upon DNA damage.

### 3.8 Functional evaluation of the mutant FANCB cDNA reveals absence of FANCD2 ubiquitination

**Figure 6:**
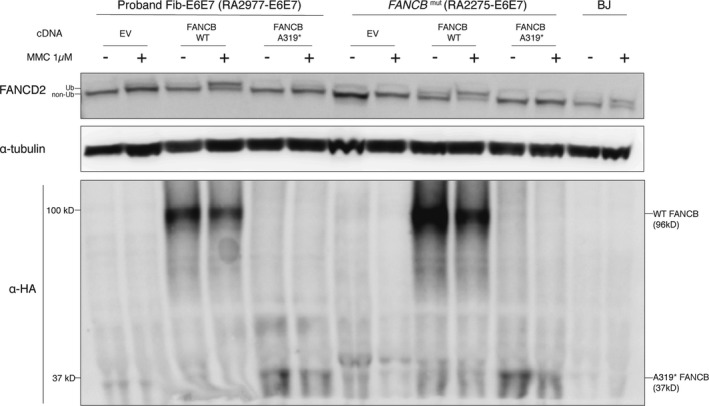
Functional evaluation of *FANCB* WT and mutant cDNA. Proband fibroblasts, FANCB mutant (null) fibroblasts and BJ control fibroblasts were HPV16 E6E7 transformed. Either empty vector, wild‐type *FANCB* cDNA, or mutant *FANCB* cDNA (p.A319*) was introduced into proband fibroblasts and FANCB mutant (null) fibroblasts. After puromycin selection, cells were cultured with or without MMC 1 μM for 24 hr, after which cells were harvested for the FANCD2 and HA western blot assays

Complementation of HPV16 E6E7‐transformed proband fibroblasts with FANCB WT cDNA restored FANCD2 ubiquitination, whereas the expression of the mutant cDNA (FANCB p.A319*, predicted to be 37kD) failed to complement FANCD2 ubiquitination upon MMC treatment (Figure 6). The expression of pA319* allele was lower than the WT suggesting that this allele was unstable. Similar complementation results were observed in an independent FANCB mutant (null) fibroblast cell line (Figure 6). We were unable to visualize the endogenous FANCB protein with the available antibodies even in the WT cells.

## 4 DISCUSSION

We describe here an FA patient assigned to the FA‐B group based on complementation tests performed on cultured skin fibroblasts. A disease‐causing intragenic duplication in *FANCB* was discovered by aCGH. Next‐gen targeted capturing and sequencing of the entire *FANCB* gene revealed the precise region of duplication. This was possible due to the high depth (~500×), complete coverage of the gene sequence in our design. The sequence reads spanning the duplication breakpoints provided the junction sequences and indicated a tandem duplication. The sequence at both ends of the duplication fragment carried a 4 bp microhomology region and the breakpoint junction retained one of these repeats. A homology of 2‐100 bp with no insertions or deletions at the breakpoint defines alternative end‐joining (alt‐EJ) (Yang et al., 2013) as a mechanism that led to the intragenic duplication. In a genome‐wide CNV analysis, it was observed that two‐thirds of genomic duplications share a short microhomology between the two ends of the duplication, and most interstitial duplications are tandem and lie in direct orientation (Newman, Hermetz, Weckselblatt, & Rudd, 2015), similar to the duplication in our FA‐B patient.

FA‐B group patients are male and either have a de novo mutation or inherit a pathogenic variant allele from a heterozygous carrier mother, as seen in our patient. Interestingly, the variant allele in the mother's PB and LCL cells was present at a lower level (21%–25%) than the 50% expected of a typical heterozygous carrier, indicating that reversions occurred even in the carrier mother. Another possibility is that the duplication occurred de novo in the mother. However, given the instability of the duplication even in the proband's fibroblasts, we favor the reversion as a source of the mosaicism in the mother. Our finding raises a possibility that some of the *FANCB* mutations ascribed to be of de novo origin in other patients may actually be present in the mother but may be mosaic in her blood. Such low‐level mosaicism may not be detected by standard testing, and thus sensitive methods should be employed to detect the variant in the carrier, prior to declaring a patient harboring a de novo variant.

The presence of mosaicism in fibroblasts (8% wild‐type allele) is unprecedented and indicative of the very unstable nature of the identified duplication. Although this low‐level mosaicism did not lead to a detectable FANCD2 ubiquitination in the primary fibroblasts, transformation of fibroblasts by HPV16 E6E7 made this mosaicism more detectable, as shown by the presence of FANCD2 ubiquitination and foci formation upon MMC exposure, presumably due to an increased selective advantage of cells with active FA pathway. We observed a 20% reversal to wild‐type in the proband LCL‐A cells grown in 2005, whereas both LCL‐A and LCL‐B cells grown in 2017 showed nearly 100% reversal to wild‐type. The LCLs show that the reversal is occurring in vitro; however, we also observed the reversal occurring in vivo, with a 90% reversal to WT in PB between 2005 and 2016. We have previously described mosaicism in an FA patient carrying an intragenic duplication of a similar length, in the *FANCT/UBE2T* (Rickman et al., 2015). That patient had variable levels of duplication in the PB cells in the *UBE2T* gene on chromosome 1. The duplication and the associated reversal were mediated by *Alu* repeats present at the ends of the duplication with nearly 90% identity in their sequences. The X‐linked *FANCB* mosaicism in our patient is interesting in that the flanking sequences share a homology of only four bases, and the reversion of the duplicated allele to WT allele may also be caused by the same microhomology‐mediated mechanism that led to the initial duplication.

Unlike typical X‐linked FA‐B patients, who usually present with severe developmental abnormalities, overlapping with the phenotype of the VACTERL syndrome with hydrocephalus, the congenital abnormalities displayed by this patient were much milder. We speculate that this could be due to the reversal of the duplication resulting in a wild‐type, functional allele in a fraction of the patient cells during fetal development. The consequence of mosaicism is apparent as the expression of WT and mutant transcripts in the RNA are proportional to the extent of duplication in the genomic DNA, suggesting there is no degradation of the aberrant transcript by nonsense‐mediated decay. However, the functional evaluation of the mutant *FANCB* cDNA (p.A319*) resulted in no FANCD2 ubiquitination, indicating that the truncated protein does not restore normal FA pathway function. *FANCB*, along with *FANCL* and *FAAP100*, is absolutely required for monoubiquitination of the *FANCD2/FANCI* complex (Huang et al., 2014; Rajendra et al., 2014), a key step that results in the recognition and initiation of DNA damage repair caused by DNA crosslinks. *FANCB* knockout mice demonstrate impairment of hematopoietic stem cell function (Du et al., 2015), one of the hallmarks of FA. Thus, molecular characterization of *FANCB* mutations and their influence on the phenotypic presentation of the patients harboring them, is critical for understanding the pathogenesis in FA.

Unlike sequence variants, duplications are difficult to identify by sequencing and may be missed. The consequences of the duplications are difficult to assess as well, unless the breakpoints are sequenced. Targeted sequencing strategies focusing only on exonic regions might not be able to provide the precise breakpoints. Conventional means of sequencing DNA and cDNA from the patient did not reveal the disease‐causing variant. This is particularly challenging when the duplication is in a mosaic form, present in only a subset of the patient cells (Biesecker & Spinner, 2013). We identified an intragenic duplication in an FA patient from an X‐linked FA‐B group by aCGH, determined the precise location of the duplication by deep sequencing, using NGS of genomic DNA, and evaluated its consequence on the transcript by PacBio sequencing. PacBio sequencing allowed sequencing larger molecules such as the 3.9 kb *FANCB* aberrant message at a higher depth, nearly 50,000 full‐length reads. This allows for detection of minute fractions of alternatively spliced products. We evaluated the biological consequence of the duplication by detailed analysis of DNA and RNA. We could detect traces of duplication even in the LCL that carried only 3% of the duplication. Thus, sensitive and versatile technologies are important to ensure proper molecular diagnosis in FA patients.

## CONFLICT OF INTEREST

None declared.
