# Supplementary material for: Somatic mosaicism of an intragenic FANCB duplication in both fibroblast and peripheral blood cells observed in a Fanconi anemia patient leads to milder phenotype
Source: Mol Genet Genomic Med. 2017 Nov 30;6(1):77–91. doi: 10.1002/mgg3.350 (PMC5823675; doi:10.1002/mgg3.350)
Supplement: Supplementary file 1 [file MGG3-6-77-s001.docx]

**Supplemental Table 1:** Diepoxybutane (DEB) breakage test for diagnosis of Fanconi anemia

| **Clinical Testing (Fig 1A)** | Proband  Fibro-2005  (RA2977) | | | Proband  PB-2005  (B05-0428.1)* | |
| --- | --- | --- | --- | --- | --- |
| DEB concentration (μg/mL) | Untreated | 0.01 | 0.1 | Untreated | 0.1 |
| # Metaphases | 96 | 85 | 19 | 50 | 50 |
| # Total breaks^+^ | 7 | 14 | 86 | 19 | 75 |
| % Metaphases with breaks | 5.2 | 14.1 | 63.2 | 26 | 30 |
| # Breaks per metaphase | 0.07 | 0.16 | 4.5 | 0.38 | 1.5 |

| **Research Testing (Fig 1B)** | *FANCA^mut^* LCL (RA2939) | | Proband  LCL-A  (RA2945) | | Proband  PB-2016 (RB16-0428.1)* | Proband  LCL-B  (RA3567) | | Mother  LCL-2016  (RA3568) | |
| --- | --- | --- | --- | --- | --- | --- | --- | --- | --- |
| DEB concentration (μg/mL) | Untreated | 0.1 | Untreated | 0.1 | 0.1 | Untreated | 0.1 | Untreated | 0.1 |
| # Metaphases | 50 | 14 | 50 | 49 | 92 | 52 | 57 | 49 | 44 |
| # Total breaks^+^ | 14 | 145 | 6 | 40 | 1 | 7 | 16 | 1 | 7 |
| % Metaphases with breaks | 22 | 100 | 10 | 32.7 | 1 | 1.9 | 12.3 | 2 | 13.6 |
| # Breaks per metaphase | 0.28 | 10.4 | 0.12 | 0.82 | 0.01 | 0.13 | 0.28 | 0.02 | 0.16 |

^+^ Total number of breaks includes chromatid breaks and radial chromosomes

^*^ Tested Independently
